# Serological Evidence of Hepatitis E Virus Infection in Brazilian Equines

**DOI:** 10.3390/microorganisms11112743

**Published:** 2023-11-10

**Authors:** Caroline Roberta Soares Salgado, Aldaleia do Nascimento e Silva, Igor Falco Arruda, Patrícia Riddell Millar, Maria Regina Reis Amendoeira, Luciane Almeida Amado Leon, Raffaella Bertoni Cavalcanti Teixeira, Jorge Tiburcio Barbosa de Lima, Flávia Löwen Levy Chalhoub, Ana Maria Bispo de Filippis, Ana Beatriz Monteiro Fonseca, Jaqueline Mendes de Oliveira, Marcelo Alves Pinto, Andreza Soriano Figueiredo

**Affiliations:** 1 Laboratório de Desenvolvimento Tecnológico em Virologia, Instituto Oswaldo Cruz, Fundação Oswaldo Cruz—Fiocruz, Rio de Janeiro 21040-900, RJ, Brazil; carolinesalgado@aluno.fiocruz.br (C.R.S.S.); jackie@ioc.fiocruz.br (J.M.d.O.); 2 Laboratório de Toxoplasmose e outras Protozooses, Instituto Oswaldo Cruz, Fundação Oswaldo Cruz—Fiocruz, Rio de Janeiro 21040-900, RJ, Brazil; igor.arruda@ioc.fiocruz.br (I.F.A.);; 3 Clínica de Grandes Animais, Departamento de Medicina Veterinária, Universidade Federal de Viçosa—UFV, Viçosa 36570-900, MG, Brazil; teixeiraraffa@gmail.com; 4 Departamento de Clínica e Cirurgia Veterinárias, Universidade Federal de Minas Gerais—UFMG, Belo Horizonte 31270-901, MG, Brazil; 5 Laboratório de Arbovírus e Vírus Hemorrágicos, Instituto Oswaldo Cruz, Fundação Oswaldo Cruz—Fiocruz, Rio de Janeiro 21040-900, RJ, Brazil; 6 Departamento de Estatística, Instituto de Matemática e Estatística, Universidade Federal Fluminense—UFF, Niterói 24210-346, RJ, Brazil

**Keywords:** hepatitis E virus, horses, equine anti-HEV IgG, zoonosis

## Abstract

Hepatitis E virus (HEV) infection has been demonstrated in various animal species; those recognized as potential zoonotic reservoirs pose a considerable risk to public health. In Brazil, HEV-3 is the only genotype identified in humans and swine nationwide, in a colony-breeding cynomolgus monkey and, recently, in bovines and capybara. There is no information regarding HEV exposure in the equine population in Brazil. This study aimed to investigate anti-HEV antibodies and viral RNA in serum samples from horses slaughtered for meat export and those bred for sport/reproduction purposes. We used a commercially available ELISA kit modified to detect species-specific anti-HEV, using an anti-horse IgG-peroxidase conjugate and evaluating different cutoff formulas and assay precision. Serum samples (n = 257) were tested for anti-HEV IgG and HEV RNA by nested RT-PCR and RT-qPCR. The overall anti-HEV seroprevalence was 26.5% (68/257) without the detection of HEV RNA. Most municipalities (53.3%) and farms (58.8%) had positive horses. Animals slaughtered for human consumption had higher risk of HEV exposure (45.5%) than those bred for sports or reproduction (6.4%) (*p* < 0.0001). The statistical analysis revealed sex and breeding system as possible risk-associated factors. The first serological evidence of HEV circulation in Brazilian equines reinforces the need for the surveillance of HEV host expansion in a one-health approach.

## 1. Introduction

Hepatitis E virus (HEV), *Paslahepevirus balayani*, is a single-strand positive-sense RNA virus that is classified into two subfamilies of the family *Hepeviridae*: *Orthohepevirinae*, including the genera *Paslahepevirus*, *Avihepevirus*, *Rocahepevirus* and *Chirohepevirus* and *Parahepevirinae*, including the genus *Piscihepevirus*. Because of their potential to cross species barriers and infect humans, *Paslahepevirus balayani* have become a public health concern worldwide. Of the eight genotypes (from HEV-1 to HEV-8) identified so far [1], HEV-1 and HEV-2, which are transmitted through the oral–fecal route, infect humans exclusively and are often associated with waterborne outbreaks of acute hepatitis in developing countries. HEV-3, HEV-4 and HEV-7 are zoonotic and are mainly transmitted through the consumption of raw or undercooked animal products. HEV-3 is associated with hepatitis cases in high-income and developing countries. Other potentially zoonotic hosts have been reported, such as horses, cattle, goats, sheep, dogs, rodents, rabbits and camelids [2,3,4]. HEV-5 and HEV-6 were described in wild boars from Japan [5]. HEV-7 was described in dromedary camels from the Middle East, and its zoonotic transmission was confirmed in an immunocompromised transplant patient [6,7]. HEV-8 was described in farmed camels from China [8].

The epidemiology of HEV in Brazil is considered similar to that of high-income countries, where HEV-3 circulates as a silent (mainly asymptomatic) infection among swine and human populations. The anti-HEV detection rates in the general population and blood donors are heterogeneous according to geographic regions, from the lowest in the North and Northeast regions (below 1%) to the highest rates in the Southeast (between 10% and 20%) and South (above 40%), in which the largest swine herds in Brazil are concentrated and where the consumption of pork products is more frequent [9,10]. The temporal distribution of anti-HEV seroprevalence has shown an increasing trend in Brazil [9], with the highest seroprevalence in blood donors from the South (up to 65%), suggesting that HEV might be highly endemic in this region [11].

HEV is present in reservoir animals in Brazil, from large- to family-scale pig farms and in wild boars. The detection of anti-HEV antibodies and HEV RNA ranges from 0% to 81.3% and from 0.84% to 87.5%, respectively, according to the geographical region and biological sample analyzed [10]. HEV RNA was also detected in 36% of pork and pork-derived products [12]. The human HEV-3 isolates, including the first autochthonous human case reported in the country [13], are closely related to swine HEV strains from the same region, thus confirming their zoonotic origin [14]. Studies investigating HEV infection in animals and other potential animal reservoirs in Brazil other than swine have demonstrated serological evidence of the viral exposure of cattle, wild rodents, dogs and chickens [15]. The HEV-3 genome was detected in the liver of slaughtered cattle [16] and in fecal samples from capybaras [17].

The expanding host range and the recent increase in human infections associated with zoonotic HEV-3 in some countries (e.g., in the European Union and Brazil) have highlighted the possible contribution of other susceptible animals to HEV transmission, variously via the consumption of raw or undercooked meat, direct contact or occupational means [9,18,19].

Horses have been shown to be susceptible to HEV infection since 2007, and their contribution as natural reservoirs has been evaluated in recent years. Their epidemiological role in zoonotic transmission cannot be ruled out since contact with horses was significantly associated with a higher risk of exposure to HEV in Denmark [20], and anti-HEV and RNA detection rates ranging from 11% to 16.3% and 0% to 4% have been reported in horses from Africa, Asia and Europe, respectively [21,22,23,24]. Brazil holds one of the largest horse populations in the world, with about 6 million animals [25] used for work, sports, reproduction, pets and meat production (the second largest in South America) [26,27]. In contrast, there is a lack of information regarding the circulation of HEV in the equine population of Brazil and the possible contribution of these animals to the spread of HEV. This study aimed to investigate the exposure of Brazilian horses to HEV infection.

## 2. Materials and Methods

### 2.1. Study Population

The population investigated was composed of 257 horses (*Equus ferus caballus*) from three Brazilian states: Bahia (BA), Goiás (GO) and Rio de Janeiro (RJ). Serum samples were grouped according to the horses’ breeding purposes.

Group A (n = 132) comprised horses from farms located in three municipalities (Cachoeira Alta, GO, n = 61; Cumari, GO, n = 49; and Serra Dourada, BA, n = 22), bred for the production of meat and milk or sports. Serum samples were obtained in 2015 in a slaughterhouse licensed by the Federal Sanitary Inspection Service for meat export which was located in the Triângulo Mineiro/Alto Paranaíba mesoregion, in the state of Minas Gerais. The blood collections were approved by the Ethics Committee for Animal Use of the Fluminense Federal University in Rio de Janeiro (CEUA UFF license 20/13). Epidemiological variables such as sex, animal origin, contact with other animals, purpose and water source were collected as described before [28] (Table 1).

Group B (n = 125) comprised horses from farms located in 12 municipalities (Araruama, Areal, Barra do Piraí, Bom Jesus do Itabapuana, Cachoeiras de Macacu, Canta Galo, Casimiro de Abreu, Duque de Caxias, Itaperuna, Macaé, Paraíba do Sul and Saquarema) from the six mesoregions of the state of Rio de Janeiro (Baixadas, Central, Metropolitan, North, Northwest, and South) which were bred for sports and reproduction. The animals were randomly sampled from January 2015 to October 2016. The blood collections were approved by the Ethics Committee on Animal Use of Oswaldo Cruz Institute–Fiocruz (CEUA IOC license 047/2015). The epidemiological variables age, sex, breed, purpose, animal origin and breeding system were collected as described before [29] (Table 1).

### 2.2. Serological Analysis

A serological investigation was conducted using a commercial human anti-HEV IgG detection kit (*recom*Well HEV IgG, Mikrogen Diagnostik, Neuried, Germany) composed of purified HEV antigens of the genotypes HEV-1 and HEV-3. The human anti-HEV IgG positive and negative controls provided with the kit were included in each run to evaluate the reaction conditions and reagents. Horse anti-HEV IgG antibodies were detected using a goat anti-horse IgG horseradish peroxidase conjugate (ab102396, ABCAM, Cambridge, UK), according to the manufacturer’s instructions with modifications. Briefly, the horse serum and assay controls were diluted in a dilution buffer to 1:100 (100 µL) and incubated for 60 min at 37 °C. After four washes, the anti-horse IgG conjugate was diluted to 1:10,000 and the anti-human IgG conjugate was diluted to 1:100 in a dilution buffer and added (100 µL) to the respective sample wells, following incubation for 30 min at 37 °C. After four washes and incubation in a tetramethylbenzidine (TMB) substrate (100 µL) for 30 min at room temperature (RT), a phosphoric acid (H_3_PO_4_) stop solution (100 µL) was added, and the optical density (OD) was read at 450 nm (630 nm reference). Horse serum samples from 18 newborn and colostrum-deprived foals (Ethics Committee on Animal Use of the Minas Gerais Federal University (CEUA UFMG license 374/2019) were used as negative controls.

The precision of the adapted assay (intra-assay) was calculated via the coefficient of variation (CV) (%) of 10 samples from Group A, 10 samples from Group B and 10 samples from newborns and colostrum-deprived foals in one assay in triplicate.

To determine the cutoff values, five methodologies (four formulas and one statistical algorithm [30]) were evaluated:

(a) Cutoff value = mean OD + 3 × standard deviation (SD) of the horse negative samples [30], where the horse negative samples were 18 serum samples derived from newborn and colostrum-deprived animals;
(b) Cutoff value = mean OD + 3 × SD of the horse negative samples, where the horse negative samples were determined according to parameters of the kit (OD < mean OD of cutoff controls) (adapted formula from [30]);
(c) Cutoff value = 2 × mean OD of the horse negative samples, where the horse negative samples were determined according to parameters of the kit (OD < mean OD of cutoff controls) (adapted formula from [30]);
(d) Cutoff value = 3 × mean OD of the horse negative samples, where the horse negative samples were determined according to parameters of the kit (OD < mean OD of cutoff controls) (adapted formula from [30]);
(e) Change-point analysis, a statistical algorithm in the R package which finds the step in the series of OD values that separates positive and negative samples [30].

### 2.3. Molecular Analysis

A molecular investigation was performed as follows. After RNA was extracted using the Relia Prep Viral Total Nucleic Acid Purification Kit (Promega, Madison, WI, USA) according to the manufacturer’s instructions, an HEV RNA amplification reaction was carried out via two strategies: (a) A quantitative reverse transcription polymerase chain reaction (RT-qPCR) with primers and a probe directed to the open reading frame-3 region (ORF-3) [31], using the SuperScript III One-Step RT-qPCR System (Invitrogen, Waltham, MA, USA) and cycling conditions according to the manufacturer’s instructions. A synthetic construct of 128 base pairs (bp) (gBlock, Promega, Madison, WI, USA) containing a conserved region among the HEV genotypes HEV-1 to HEV-4 and an HEV-RNA-genotype-HEV-3-positive fecal sample [32] were included as positive controls. (b) A nested RT-PCR with primers directed to the ORF-2 region (436 bp’s) [22] (final concentration: 0.5 µM) using SuperScript III and Platinum Taq DNA Polymerase kits (Invitrogen, Waltham, MA, USA) with an annealing temperature of 50 °C for 30 s in the first and second rounds. The HEV-RNA-genotype-3-positive fecal sample described above was nucleotide-sequenced (436 bp’s) (RPT01A Plataforma de Sequenciamento, Fiocruz) and deposited in the GenBank with accession number OQ728817 (SisGen number AD69978).

### 2.4. Statistical Analysis

Descriptive and statistical analyses were performed to evaluate the serological results and epidemiological variables. Cohen’s Kappa coefficient was employed to compare, in each assay, the cutoff values defined by the methodologies (b), (c) and (d) (Section 2.2) with those defined by Classification and Regression Tree (CART)-built Chi-squared Automatic Interaction Detection (CHAID). The associations between two categorical variables were determined using Fisher’s exact test. A forward stepwise logistic regression with Wald’s test for model selection was used to evaluate possible risk factors associated with the detection of anti-HEV antibodies. “OD” and “Age” were analyzed using the Mann–Whitney or Kruskal–Wallis tests when appropriate. The associations between these variables were analyzed using the Pearson correlation coefficient. The odds ratio (OR) and 95% confidence interval (CI 95%) of the OR were determined. The global significance level adopted was 5%.

## 3. Results

### 3.1. Serological Evidence of HEV Infection

#### 3.1.1. Evaluation of the Adapted ELISA Assay and Cutoff Determination

**Figure 1:**
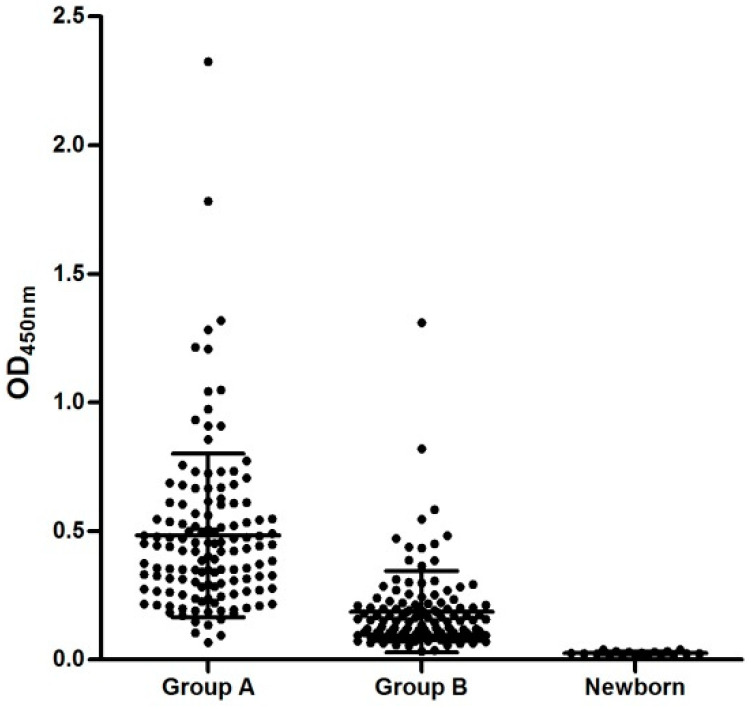
Graphical presentation of OD values obtained in each group of animals (means ± SD) (*p* < 0.0001) tested with an adapted equine anti-HEV antibody assay.

The horse anti-HEV IgG assay performed with all serum samples presented an OD varying from 0.018 to 2.325, with a mean ± standard deviation (SD) of 0.318 ± 0.29. The quantitative variables “OD” and “Age” had no correlation (Pearson correlation of −0.025, *p* = 0.786). The OD variations according to animal groups are presented in Table 1 and Figure 1.

To evaluate the intra-assay variation, ten samples from each animal group were tested in triplicate, with an overall CV of 10.03%. The CV obtained in each group is presented in Table 2.

To discriminate anti-HEV-IgG-positive and -negative samples, five methodologies for calculating cutoffs were evaluated (Table 3). Methodologies (a) and (d) presented the lowest (0.0443) and highest (mean: 0.670) cutoff values, respectively. Methodologies (b) and (c) presented similar cutoff values (means of 0.469 and 0.447, respectively). The statistical algorithm of methodology (e) did not find the step in the series of OD values that separated the positive and negative samples.

The samples from newborn/colostrum-deprived foals resulted in a low cutoff value (they are known to have low or absent levels of immune proteins [33]), with a consequent overestimation of the detection of anti-HEV IgG antibodies when formula (a) was employed (>99%). Considering that formula (d) was previously described to underestimate detection rates, and formulas (b) and (c) could give some false positive results, with a final recommendation of formula (b) [2], this formula was chosen for further analysis. To test the reliability of each of the four cutoff values obtained via the methodologies (b), (c) and (d), four classification and regression trees (CARTs) were calculated and compared using Cohen’s Kappa statistical coefficient. The results indicated a high level of agreement, with Kappa > 0.935 for methodology (b) (Table 3).

#### 3.1.2. Descriptive and Statistical Analyses

The overall rate of detection of anti-HEV antibodies was 26.5% (68/257): 45.5% (60/132) in Group A and 6.4% (8/125) in Group B (Table 4). Regarding the sampling location, 53.3% (8/15) of the municipalities and 58.8% (10/17) of the farms had positive animals. The general epidemiological information about the total population studied and the relative frequency of the detection of anti-HEV antibodies are presented in Table 4.

**Figure 2:**
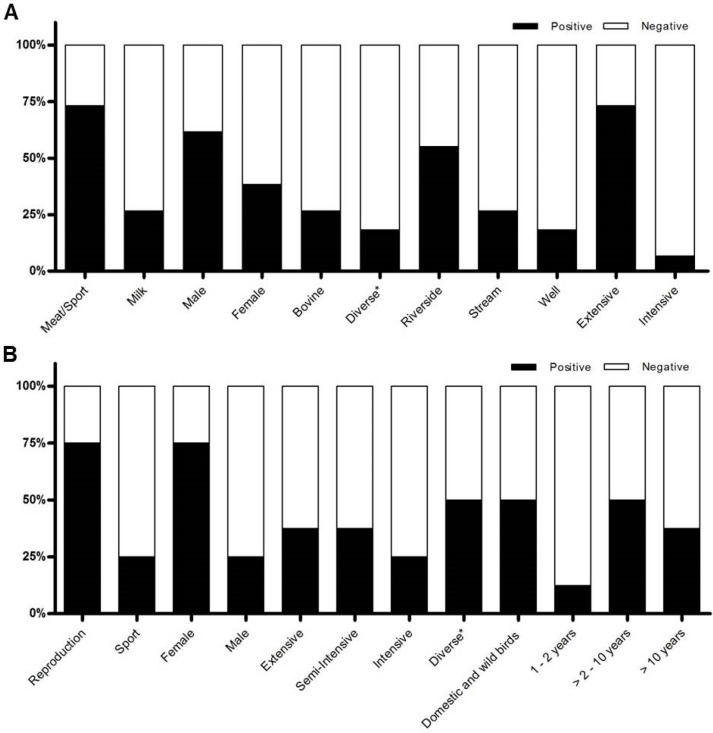
Graphical presentation of positive rates of detection of horse anti-HEV IgG antibodies (%) according to the epidemiological variables of Groups A (**A**) and B (**B**).

The rates of positive results for anti-HEV antibodies according to the epidemiological information available for the individual groups are presented in Figure 2. With respect to the common epidemiological variables between groups, most of the positive horses were males (57.3%, 39/68), and most of the positive animals were raised in extensive systems (69.1%, 47/68). Among the positive animals, 45.6% (31/68) were raised in contact with pigs and other animals, such as cattle.

The statistical analysis showed a high risk of HEV exposure among animals slaughtered for meat export (Group A) (*p* < 0.0001). The variables “Sex” (*p* = 0.0228) and “Breeding system” were also risk-associated factors (*p* = 0.0321). Other epidemiological variables were not statistically significant (Table 5). “Group” was the only retained variable (*p* < 0.0001) after a logistic regression analysis of possible risk factors, with Group A presenting an OR of 2.5 (CI 95% 1.504 to 4.173).

### 3.2. Molecular Investigation of HEV Infection

HEV RNA was neither detected via RT-qPCR nor nested PCR (designed for ORF-3 and ORF-2 genome regions, respectively).

## 4. Discussion

This study provides the first serological evidence of HEV infection in Brazilian horses. Horse-specific anti-HEV IgG antibodies were detected in two equine cohorts grouped according to breeding purposes. The overall antibody detection rate of 26.5% (68/257) was higher than those reported in China (16.3%) [34], Egypt (13.0%) [23], South Korea (12.4%) [24] and the Netherlands (18.2%) [35]. However, data from studies using different detection strategies and equine cohorts cannot be directly compared. For example, the first study of HEV exposure in horses, dating from the late 2000s, used a commercial ELISA based on HEV-1 ORF-2 recombinant proteins and modified with an anti-horse secondary antibody to detect anti-HEV IgG (specificity confirmed via an inhibition assay) [34]. In contrast, further studies used the species-independent double-antigen sandwich ELISA, either developed in-house or commercially available, which were also based on HEV-1 ORF-2 recombinant proteins and are able to detect total anti-HEV antibodies (IgG, IgM and IgA) [24,35,36]. As an alternative to multispecies anti-HEV assays, we adapted a commercially available human anti-HEV ELISA kit based on HEV-1 and HEV-3 recombinant proteins to detect horse-specific anti-HEV IgG by replacing the human anti-IgG conjugate with a species-specific secondary antibody. This strategy was also employed for anti-HEV detection in hogs [37] and donkeys [22]. The reliability and consistency of the results obtained with the modified ELISA were assured, as the controls provided by the manufacturer were validated in every run accordingly. The test precision, ascertained by the intra-assay coefficient of variation, presented acceptable levels (mean CV < 15%) [38]. Serum samples obtained from colostrum-deprived newborn foals (n = 18) were used as negative controls (a strategy also employed for donkeys [22]) since there is no transference of immunoglobulins through the diffuse epitheliochorial placenta of the mares [33]. However, when applied to the formula cutoff = mean of negative controls + 3 × SD [39,40,41], the mean optical density (OD 0.0263 ± 0.006) obtained for the foal’s sera resulted in an overestimated anti-HEV IgG detection rate of more than 99% positivity (up to 18% in previous studies) [22,23,34,35]). The challenge of setting a cutoff value without known negative and positive samples could be overcome by applying the changing-point statistical algorithm, which does not require predefined samples for calculation. Unfortunately, the OD values did not present a step in the series to discriminate between positive and negative samples. The alternative was to evaluate different cutoff formulas [30], adapting them to use equine negative samples classified according to the manufacturer’s parameters, as described previously [37], and setting the formula after the statistical evaluation.

Whether the heterogeneous anti-HEV seroprevalence data reported in different studies can be attributed to sensitivity and specificity nuances between the immunoassays employed is still controversial. Moreover, this issue is also a concern when different commercial ELISA kits are used in human and swine seroprevalence studies [9,42,43]. Beyond the detection rates, our findings evidenced a higher risk of exposure to HEV (*p* < 0.0001) that was about two to five times greater in horses slaughtered for human consumption than in horses bred for sports and reproduction purposes; this result was supported by both binomial and logistic regression statistical analyses. In Brazil, the equid meat market is almost solely intended for exportation, considering the insignificant portion represented by the domestic Brazilian trade. It is characterized by a lack of a production system, with legal approaches directed only to slaughter, processing and distribution. The equids sent to slaughterhouses are, usually, those not considered appropriate for their primary purposes and are old animals or animals presenting health issues. They no longer promptly receive veterinary assistance nor have tracking records, which does not favor their sanitary conditions nor animal welfare [44,45]. These are issues that have already raised the concern of the risks of infection with other etiological agents transmitted via the consumption of raw or undercooked equid meat, such as *Toxoplasma gondii* [28,44,45].

The statistical analysis revealed that the breeding system (*p* = 0.0321) is a possible risk factor associated with the detection of anti-HEV antibodies. Most anti-HEV positive animals were raised in the extensive system (69.1%), which may favor HEV exposure via contact with other susceptible and reservoir animals raised together, as well as contact with contaminated pastures. This hypothesis was also reported for racing horses [24] when the results suggested that their breeding environment could be exposed to HEV reservoirs and contaminated with HEV. Studies conducted with hybrid pigs and domestic pig farms demonstrated that the rates of HEV exposure were associated with different breeding environments and systems, especially with regard to the presence of other animal species and possible environmental contaminants [46,47,48]. In Brazil, the co-grazing of horses with other animals is practiced nationwide, with benefits for the ecosystem [49] and improvements in vegetation and animal health (e.g., decreasing parasitism) [50]. In this study, a large proportion of the investigated animals were bred together with mules, donkeys, cattle, hogs and/or other animals (45.6%), further supporting the possible contribution of the breeding environment to HEV exposure.

The age of the horses was not considered a risk factor (*p* = 0.757) in this study. However, the small number of animals with a documented age among those who were anti-HEV-positive (8/68) may have hampered the statistical evaluation. It is noteworthy that a positive correlation between age and HEV seroprevalence is well documented in human studies [51,52] and was also demonstrated in donkeys bred for meat in China [22]. Considering that the horses slaughtered are usually old animals [44,45], this may also contribute to the higher rate of anti-HEV antibody detection demonstrated in this group (*p* < 0.0001). This could also be true for the absence of HEV RNA detection in this study, since age was inversely correlated with RNA positivity in the study conducted in Chinese donkeys [22], but age was also not significantly associated with the detection of anti-HEV antibodies in a study from South Korea [24], and contrasting findings demonstrated higher rates of viral RNA detection in old horses from Spain [21].

In contrast to other studies, the epidemiological variable sex was significantly associated with the detection of anti-HEV antibodies in this study (*p* = 0.0228), with males presenting an approximately 1.9-fold greater chance of exposure to HEV than females. This might be due to the high number of male horses employed in the activities of Group A which are raised in the extensive system (Figure 1) in contact with other susceptible animals.

In conclusion, this study demonstrates for the first time the serological evidence of HEV exposure in the Brazilian horse population. Although the viral genome was not detected, an important proportion of horses slaughtered for human consumption had contact with the virus, which poses a challenge to researchers and authorities to further investigate the potential of horses as HEV reservoirs and possible routes of the transmission of HEV to humans and other animals. Nonetheless, further studies are needed to assess other potential risk factors and to confirm the prevalence of HEV infection in Brazilian horses.

## Figures and Tables

**Table 1:** OD values obtained via the horse anti-HEV IgG assay.

OD Value	Mean ± SD	*p* Value	CI 95%
Group A	0.483 ± 0.317	<0.0001	0.428 to 0.538
Group B	0.186 ± 0.158		0.158 to 0.214
Newborn/colostrum-deprived	0.0263 ± 0.006		0.0231 to 0.0295

**Table 2:** Coefficients of variation of the intra-assay evaluation.

Sample	Mean ± SD	CV (%)
Group A (n = 10)	0.463 ± 0.041	9.5
Group B (n = 10)	0.271 ± 0.024	8.5
Newborn/colostrum-deprived foals (n = 10)	0.017 ± 0.002	12.1
Mean		10.03

**Table 3:** Evaluation of cutoff values (methodology (b), in bold, was chosen for further epidemiological and statistical analyses).

	Cutoff ^1^	Group A Positive (%)	Group B Positive (%)	Overall % of Positive	Group A × B	OR	CI 95%	Cohen’s Kappa
(a)	0.0443	132 (100)	123 (98.4)	99.22	-	-	-	-
**(b)**	**0.608**	**60 (45.5)**	**08 (6.4)**	**26.46**	***p* < 0.0001**	**12.19**	**5.51 to 26.97**	1.0
	**0.427**							0.977
	**0.354**							0.935
	**0.488**							0.959
(c)	0.677	54 (40.9)	12 (9.6)	25.68	*p* < 0.0001	6.52	3.273 to 12.98	0.857
	0.460							1.0
	0.305							1.0
	0.345							1.0
(d)	1.016	27 (20.5)	05 (4.0)	12.45	*p* < 0.0001	6.17	2.294 to 16.60	1.0
	0.689							0.863
	0.458							0.693
	0.517							0.954
(e)	Failed	-	-	-	-	-	-	-

^1^ Formulas (b), (c) and (d) had cutoff calculations for each assay performed (total of 4 assays).

**Table 4:** Epidemiological information about the total population studied (n = 257) and the relative frequency of the detection of anti-HEV IgG antibodies.

Variables	N (%)	Relative Frequency (%)
Purpose ^1^		
Meat	61 (23.7)	54.1
Milk	49 (19.1)	32.7
Reproduction	44 (17.1)	4.5
Sport	81 (31.5)	7.4
Sport/Meat	22 (8.7)	50.0
Age (years) ^2^		
0–2	27 (10.5)	3.7
>2–5	28 (10.9)	14.3
>5–10	36 (14.0)	2.8
>10–15	12 (4.7)	16.7
>15	22 (8.6)	0.0
NI	132 (51.4)	45.5
Sex		
Female	141 (54.8)	20.6
Male	116 (45.2)	33.6
Breed ^2^		
MM	65 (25.3)	6.2
Campolina	53 (20.6)	5.7
Quarter Horse	4 (1.6)	25.0
Crossbreed	3 (1.2)	0.0
NI	132 (51.4)	45.5
Other animals		
Bovine	71 (27.6)	25.4
Diverse ^3^	47 (18.3)	27.7
Only horses	94 (36.6)	35.1
Domestic and wild birds	45 (17.5)	8.9
System		
Extensive	156 (60.7)	30.1
Intensive	23 (9.0)	26.1
Semi-intensive	35 (13.6)	8.6
NI	43 (16.7)	27.9
Water source ^4^		
Riverside	61 (46.2)	54.1
Stream	49 (37.1)	32.7
Well	22 (16,7)	50.0
NI	125 (48.6)	6.4

^1^ Group A: meat, milk and sport/meat; Group B: sport and reproduction. ^2^ All animals from Group A are missing the “Age” and “Breed” data. ^3^ Diverse: bovine, swine, donkey, goat, mule, poultry and sheep. ^4^ All animals from B are missing “Water source” data. NI: not informed; MM: Mangalarga Marchador.

**Table 5:** Risk factors associated with the detection of anti-HEV IgG antibodies.

Variable	*p* Value	Odds Ratio	CI 95%
Group A × B	<0.0001	12.19	5.51 to 26.97
Purpose Group A ^1^			
Sport/Meat × Milk	0.1436	1.828	0.875 to 3.819
Purpose Group B ^1^			
Reproduction × Sport	0.7115	0.5952	0.1149 to 3.083
Breeding system			
Extensive × SI × Intensive	0.0321	-	-
Extensive × SI	0.0096	4.599	1.34 to 15.77
Sex			
Male × Female	0.0228	1.956	1.11 to 3.43
Age (years) ^2^			
1–2× > 2–10× > 10	0.757	-	-
Breed ^2^			
MM × CH × QH × Crossbreed	1.0	-	-
Other animals ^3^			
Bovine × Diverse ^4^ × None	0.0721	-	-
Water source ^3^			
Riverside × Stream × Well	0.072	-	-

^1^ The “Purpose” variable is presented by group since there were different purposes in each. ^2^ “Age” and “Breed” information are available only for Group B. ^3^ “Other animals” and “Water source” information are available only for Group A. ^4^ Diverse: bovine, swine, donkey, goat, mule, poultry and sheep.

## Data Availability

The molecular data obtained in this study are available in GenBank under the accession number OQ728817.

## References

[1] Smith D., Drexler J.F., Meng X.-J., Norder H., Okamoto H., van der Poel W.H.M., Purdy M.A., Reuter G., de Souza W.M., Ulrich R.G. Taxon Details|ICTV. https://ictv.global/taxonomy/taxondetails?taxnode_id=202203665..

[2] Spahr C., Knauf-Witzens T., Vahlenkamp T., Ulrich R.G., Johne R. (2018). Hepatitis E virus and related viruses in wild, domestic and zoo animals: A review. Zoonoses Public Health.

[3] Wang B., Meng X.J. (2021). Hepatitis E virus: Host tropism and zoonotic infection. Curr. Opin. Microbiol..

[4] Kenney S.P. (2019). The current host range of hepatitis E viruses. Viruses.

[5] Takahashi M., Nishizawa T., Sato H., Sato Y., Jirintai, Nagashima S., Okamoto H. (2011). Analysis of the full-length genome of a Hepatitis E Virus isolate obtained from a wild boar in Japan that is classifiable into a Novel Genotype. J. Gen. Virol..

[6] Rasche A., Saqib M., Liljander A.M., Bornstein S., Zohaib A., Renneker S., Steinhagen K., Wernery R., Younan M., Gluecks I. (2016). Hepatitis E virus infection in dromedaries, North and East Africa, United Arab Emirates, and Pakistan, 1983–2015. Emerg. Infect. Dis..

[7] Lee G.-H., Tan B.-H., Teo E.C.-Y., Lim S.-G., Dan Y.-Y., Wee A., Aw P.P.K., Zhu Y., Hibberd M.L., Tan C.-K. (2016). Chronic Infection with Camelid Hepatitis e Virus in a Liver Transplant Recipient Who Regularly Consumes Camel Meat and Milk. Gastroenterology.

[8] Woo P.C., Lau S.K., Teng J.L., Tsang A.K.L., Joseph M., Wong E.Y., Tang Y., Sivakumar S., Xie J., Bai R. (2014). New hepatitis E virus genotype in camels, the Middle East. Emerg. Infect. Dis..

[9] de Oliveira J.M., Santos D.R.L.D., Pinto M.A. (2023). Hepatitis E Virus Research in Brazil: Looking Back and Forwards. Viruses.

[10] Moraes D.F.d.S.D., Mesquita J.R., Dutra V., Nascimento M.S.J. (2021). Systematic review of hepatitis e virus in Brazil: A one-health approach of the human-animal-environment triad. Animals.

[11] Pandolfi R., De Almeida D.R., Pinto M.A., Kreutz L.C., Frandoloso R. (2017). In house ELISA based on recombinant ORF2 protein underline high prevalence of IgG antihepatitis e virus amongst blood donors in south Brazil. PLoS ONE.

[12] Heldt F.H., Staggmeier R., Gularte J.S., Demoliner M., Henzel A., Spilki F.R. (2016). Hepatitis E Virus in Surface Water, Sediments, and Pork Products Marketed in Southern Brazil. Food Environ. Virol..

[13] Lopes dos Santos D.R., Lewis-Ximenez L.L., da Silva M.F.M., de Sousa P.S.F., Gaspar A.M.C., Pinto M.A. (2010). First report of a human autochthonous hepatitis E virus infection in Brazil. J. Clin. Virol..

[14] Dos Santos D.R.L., Durães-Carvalho R., Gardinali N.R., Machado L.C., de Paula V.S., Wallau G.L., Oliveira J.M., Pena L.J., Pinto M.A., Gil L.H.V.G. (2023). Uncovering neglected subtypes and zoonotic transmission of Hepatitis E virus (HEV) in Brazil. Virol. J..

[15] Vitral C.L., Pinto M.A., Lewis-Ximenez L.L., Khudyakov Y.E., Santos D.R.D., Gaspar A.M.C. (2005). Serological evidence of hepatitis E virus infection in different animal species from the Southeast of Brazil. Mem. Inst. Oswaldo Cruz.

[16] Bastos C., Eisen A.K.A., Demoliner M., Heldt F.H., Filippi M., Pereira V.M.d.A.G., Teixeira T.A.M., Roth L.O., Gularte J.S., Spilki F.R. (2022). Hepatitis E virus genotype 3 in bovine livers slaughtered in the state of Rio Grande do Sul, Brazil. Braz. J. Microbiol..

[17] Cunha L., Luchs A., Azevedo L.S., Silva V.C.M., Lemos M.F., Costa A.C., Compri A.P., França Y., Viana E., Malta F. (2023). Detection of Hepatitis E Virus Genotype 3 in Feces of Capybaras (*Hydrochoeris hydrochaeris*) in Brazil. Viruses.

[18] Adlhoch C., Avellon A., Baylis S.A., Ciccaglione A.R., Couturier E., de Sousa R., Epštein J., Ethelberg S., Faber M., Fehér A. (2016). Hepatitis E virus: Assessment of the epidemiological situation in humans in Europe, 2014/15. J. Clin. Virol..

[19] Izopet J., Tremeaux P., Marion O., Migueres M., Capelli N., Chapuy-Regaud S., Mansuy J.-M., Abravanel F., Kamar N., Lhomme S. (2019). Hepatitis E virus infections in Europe. J. Clin. Virol..

[20] Christensen P.B., Engle R.E., Hjort C., Homburg K.M., Vach W., Georgsen J., Purcell R.H. (2008). Time trend of the prevalence of hepatitis E antibodies among farmers and blood donors: A potential zoonosis in Denmark. Clin. Infect. Dis..

[21] García-Bocanegra I., Rivero A., Caballero-Gómez J., López-López P., Cano-Terriza D., Frías M., Jiménez-Ruiz S., Risalde M.A., Gómez-Villamandos J.C., Rivero-Juarez A. (2019). Hepatitis E virus infection in equines in Spain. Transbound. Emerg. Dis..

[22] Rui P., Zhao F., Yan S., Wang C., Fu Q., Hao J., Zhou X., Zhong H., Tang M., Hui W. (2020). Detection of hepatitis E virus genotypes 3 and 4 in donkeys in northern China. Equine Vet. J..

[23] Saad M.D., Hussein H.A., Bashandy M.M., Kamel H.H., Earhart K.C., Fryauff D.J., Younan M., Mohamed A.H. (2007). Hepatitis E virus Infection in Work Horses in Egypt. Infect. Genet. Evol..

[24] Yoon J., Park T., Sohn Y., Park B.-J., Ahn H.-S., Go H.-J., Kim D.-H., Lee J.-B., Park S.-Y., Song C.-S. (2022). Surveillance of hepatitis E virus in the horse population of Korea: A serological and molecular approach. Infect. Genet. Evol..

[25] 2017|IBGE. https://www.ibge.gov.br/estatisticas/economicas/agricultura-e-pecuaria/21814-2017-censo-agropecuario.html.

[26] MAPA (2016). Revisão do Estudo do Complexo do Agronegócio do Cavalo.

[27] FAOSTAT. https://www.fao.org/faostat/en/#data/QCL/visualize.

[28] Arruda I.F., Freitas W.A., Carrijo K.F., Paz P.S., Silva M.M., Sudré A.P., Marques-Santos F., Fonseca A.B.M., Amendoeira M.R.R., Millar P.R. (2020). Occurrence of anti-toxoplasma gondii antibodies and risk factors associated with infection in equids slaughtered for human consumption in Brazil. Rev. Bras. Parasitol. Vet..

[29] Figueiredo A.S., Lampe E., de Albuquerque P.P.L.F., Chalhoub F.L.L., de Filippis A.M.B., Villar L.M., Cruz O.G., Pinto M.A., de Oliveira J.M. (2018). Epidemiological investigation and analysis of the NS5B gene and protein variability of non-primate hepacivirus in several horse cohorts in Rio de Janeiro state, Brazil. Infect. Genet. Evol..

[30] Lardeux F., Torrico G., Aliaga C. (2016). Calculation of the ELISA’s cut-off based on the change-point analysis method for detection of Trypanosoma cruzi infection in Bolivian dogs in the absence of controls. Mem. Inst. Oswaldo Cruz.

[31] Jothikumar N., Cromeans T.L., Robertson B.H., Meng X.J., Hill V.R. (2006). A broadly reactive one-step real-time RT-PCR assay for rapid and sensitive detection of hepatitis E virus. J. Virol. Methods.

[32] Gardinali N.R., Guimarães J.R., Gil Melgaço J., Kevorkian Y.B., Bottino F.d.O., Vieira Y.R., Silva A.C.d.A.d., Pinto D.P., da Fonseca L.B., Vilhena L.S. (2017). Cynomolgus monkeys are successfully and persistently infected with hepatitis E virus genotype 3 (HEV-3) after long-term immunosuppressive therapy. PLoS ONE.

[33] Jeffcott L.B. (1974). Some Practical Aspects of the Transfer of Passive Immunity to Newborn Foals. Equine Vet. J..

[34] Zhang W., Shen Q., Mou J., Gong G., Yang Z., Cui L., Zhu J., Ju G., Hua X. (2008). Hepatitis E virus infection among domestic animals in eastern China. Zoonoses Public Health.

[35] Li Y., Qu C., Spee B., Zhang R., Penning L.C., de Man R.A., Peppelenbosch M.P., Fieten H., Pan Q. (2020). Hepatitis e virus seroprevalence in pets in the Netherlands and the permissiveness of canine liver cells to the infection. Ir. Vet. J..

[36] Fu H., Wang L., Li Y., Du G., Zhuang H., Zhu Y., Xue C., Li L., Chang Y., Geng J. (2010). Hepatitis E virus infection among animals and humans in Xinjiang, China: Possibility of swine to human transmission of sporadic hepatitis E in an endemic area. Am. J. Trop. Med. Hyg..

[37] Yoo D., Willson P., Pei Y., Hayes M.A., Deckert A., Dewey C.E., Friendship R.M., Yoon Y., Gottschalk M., Yason C. (2001). Prevalence of Hepatitis E Virus Antibodies in Canadian Swine Herds and Identification of a Novel Variant of Swine Hepatitis E Virus. Clin. Diagn. Lab. Immunol..

[38] European Medicines Agency (2019). ICH Guideline M10 on Bioanalytical Method Validation. Sci. Med. Health.

[39] Willen L., Mertens P., Volf P. (2018). Evaluation of the rSP03B sero-strip, a newly proposed rapid test for canine 429 exposure to *Phlebotomus perniciosus*, vector of *Leishmania infantum*. PLoS Negl. Trop. Dis..

[40] Oliveira S.A.M., Brum M.C.S., Anziliero D., Dellagostin O., Weiblen R., Flores E.F. (2013). Prokaryotic 432 expression of a truncated form of bovine herpesvirus 1 glycoprotein E (gE) and its use in an ELISA for gE 433 antibodie. Pesqui. Vet. Bras..

[41] Lunn J.A., Lee R., Smaller J., MacKay B.M., King T., Hunt G.B., Martin P., Krockenberger M.B., Spielman D., Malik R. (2012). Twenty two cases of canine neural angiostronglyosis in eastern Australia (2002–2005) and a 435 review of the literature. Parasites Vectors.

[42] Pisano M.B., Campbell C., Anugwom C., Ré V.E., Debes J.D. (2022). Hepatitis E virus infection in the United States: Seroprevalence, risk factors and the influence of immunological assays. PLoS ONE.

[43] de Almeida e Araujo D.C., de Oliveira J.M., Haddad S.K., da Roza D.L., Bottino F.d.O., Faria S.B.S.C., Bellíssimo-Rodrigues F., Passos A.D.C. (2020). Declining prevalence of hepatitis A and silent circulation of hepatitis E virus infection in southeastern Brazil. IJID.

[44] Evers F., Garcia J.L., Navarro I.T., de Freitas J.C., Bonesi G.L., do Nascimento Benitez A., Nino B.S.L., Ewald M.P.C., Taroda A., Almeida J.C. (2012). Zoonosis of public health interest in slaughtered Brazilian equidae. Semin. Ciênc Agrár..

[45] Junqueira A., Bressan M.C., Rebello F.F.P., Faria P.B., Vieira J.O., Savian T.V. (2005). Composição centesimal e teor de colesterol na carne de equinos (*Equus caballus*, Linneaus, 1758) machos e fêmeas agrupados por peso de carcaça. Ciência Agrotecnologia.

[46] Jori F., Laval M., Maestrini O., Casabianca F., Charrier F., Pavio N. (2016). Assessment of domestic pigs, wild boars and feral hybrid pigs as reservoirs of hepatitis E virus in Corsica, France. Viruses.

[47] Lopez-Lopez P., Risalde M.d.L.A., Frias M., García-Bocanegra I., Brieva T., Caballero-Gomez J., Camacho A., Fernández-Molera V., Machuca I., Gomez-Villamandos J.C. (2018). Risk factors associated with hepatitis E virus in pigs from different production systems. Vet. Microbiol..

[48] Capai L., Maestrini O., Casabianca F., Villechenaud N., Masse S., Bosseur F., Lamballerie X., Charrel R.N., Falchi A. (2019). Drastic decline of hepatitis E virus detection in domestic pigs after the age of 6 months, Corsica, France. Transbound. Emerg. Dis..

[49] Baggio R., Overbeck G.E., Durigan G., Pillar V.D. (2021). To graze or not to graze: A core question for conservation and sustainable use of grassy ecosystems in Brazil. Perspect. Ecol. Conserv..

[50] Fleurance G., Sallé G., Lansade L., Wimel L., Dumont B. (2022). Comparing the effects of horse grazing alone or with cattle on horse parasitism and vegetation use in a mesophile pasture. Grass Forage Sci..

[51] da Silva C.M., Oliveira J.M., Mendoza-Sassi R.A., Figueiredo A.S., Mota L.D., Nader M.M., Gardinali N.R., Kevorkian Y.B., Salvador S.B.S., Pinto M.A. (2019). Detection and characterization of hepatitis E virus genotype 3 in HIV-infected patients and blood donors from southern Brazil. Int. J. Infect. Dis..

[52] Lewis H.C., Wichmann O., Duizer E. (2010). Transmission routes and risk factors for autochthonous hepatitis E virus infection in Europe: A systematic review. Epidemiol. Infect..

